# Quantification of noradrenergic‐, dopaminergic‐, and tectal‐neurons during aging in the short‐lived killifish *Nothobranchius furzeri*

**DOI:** 10.1111/acel.13689

**Published:** 2022-08-19

**Authors:** Sara Bagnoli, Baldassare Fronte, Carlo Bibbiani, Eva Terzibasi Tozzini, Alessandro Cellerino

**Affiliations:** ^1^ Laboratory of Biology (BIO@SNS) Scuola Normale Superiore Pisa Italy; ^2^ Department of Veterinary Sciences University of Pisa Pisa Italy; ^3^ Biology and Evolution of Marine Organisms Dep. (BEOM) Stazione Zoologica Anton Dohrn Naples Italy; ^4^ Leibniz Institute on Aging Fritz Lipmann Institute Jena Germany

**Keywords:** age‐related disease, aging, animal model, killifish, *locus coeruleus*, neurodegeneration, Parkinson's disease, teleost

## Abstract

Parkinson's disease (PD) is characterized by phosphorylation and aggregation of the protein α‐Synuclein and ensuing neuronal death progressing from the noradrenergic *locus coeruleus* to midbrain dopaminergic neurons. In 2019, Matsui and colleagues reported a spontaneous age‐dependent degeneration of dopaminergic neurons and an even greater neurodegeneration of the noradrenergic neurons in the short‐lived killifish *Nothobranchius furzeri*. Given the great possible relevance of a spontaneous model for PD, we assessed neurodegeneration of noradrenergic and dopaminergic neurons in two further laboratory strains of *N. furzeri*. We implemented, for the first time in *N. furzeri,* a whole‐brain clarification technique and proceeded to entire 3D nuclei reconstruction to quantify total cell numbers in two different stains of *N. furzeri*. In both strains, we observed that age‐dependent neurodegeneration is limited to the *locus coeruleus* and does not involve the *posterior tuberculum*. We also applied 3D counting to the optic tectum, an area of active adult neurogenesis, and detected an increase of neurons with age. Our results confirm age‐dependent neurodegeneration of noradrenergic neurons, a condition reminiscent of the presymptomatic stage of PD indicating that *N. furzeri* could be used in the future to identify modifying factors for age‐dependent neurodegeneration and open the intriguing possibility that natural genetic variation may influence the susceptibility of dopaminergic neurons.

## Graphical abstract

We used 3D reconstructions to count all tyrosine hydroxylase positive neurons in the hypothalamus and locus coeruleus of *Nothobranchius furzeri* at various ages. Our data showed presence of neurodegeneration of noradrenergic neurons in old *N. furzeri* brains restricted to the area of the locus coeruleus. No indication of neurodegeneration at any age was found in the dopaminergic neurons of the hypothalamus.

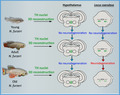

**Abbreviations**
α‐Syn: alpha‐Synuclein
LB: Lewy bodies
LC: *locus coeruleus*
LN: Lewy neurites
TH: tyrosine hydroxylase
PD: Parkinson's disease

## 1 INTRODUCTION

Parkinson's disease (PD) is one of the most prevalent neurodegenerative diseases characterized by a plethora of motor and non‐motor symptoms due to dopaminergic and noradrenergic neuron loss. It has been long hypothesized that the pathogenic mechanism of neurodegeneration lies in the misfolding and subsequent aggregation of the neuronal protein α‐Synuclein (α‐Syn), leading to the formation of intracellular Lewy bodies (LB) and dystrophic Lewy neurites (LN) (Anderson et al., 2006; Braak et al., 1999, 2002; Samuel et al., 2016; Spillantini et al., 1997). Aggregates are associated with post‐translational modifications of α‐Synuclein and in particular phosphorylation of Serine 129 (pS129, Anderson et al., 2006). Aggregation and phosphorylation appear with a precise spatio‐temporal progression that defines a staging for the pathological progression (Braak et al., 2002, 2003): The aggregation in its very early stages is observed in distal parts of the CNS and in particular the dorsal vagal nuclei in the brain stem then expands anteriorly hitting first the *locus coeruleus* (LC), subsequently the *substantia nigra* (SN) *pars compacta* causing the onset of the typical motor symptoms and ultimately affecting the most anterior parts of the cortex. Propagation of α‐Syn aggregates along the vagus nerve was recently demonstrated in animal models (Kim et al., 2019). *Locus coeruleus* and *substantia nigra* are the most studied nuclei in the context of PD. LC is affected at very early stages of PD and the extent of neuron loss is larger than in the SN, which is only affected during middle stages of the pathology (Braak et al., 2003; Zarow et al., 2003). It is also worth noting that presence of p‐Syn immunoreactivity and LB are not an exclusive feature of PD. They are found in ~10% of clinically unimpaired subjects over the age of 60 with a distribution similar to PD, but showing lesser density of inclusions and a lesser degree of tyrosine hydroxylase (TH) reduction, as compared to what observed in PD, suggesting that p‐Syn immunoreactivity and LB formation are already detectable at preclinical stages, before the onset of motor symptoms (Dickson et al., 2008; Forno, 1969; Fumimura et al., 2007).

Several approaches were undertaken to model PD in animal models. Dopaminergic loss can be induced in rodents by acute pharmacological treatments (for reviews see Meredith & Rademacher, 2011; Johnson & Bobrovskaya, 2015), but this approach fails to model the progressive and age‐associated nature of PD. An alternative approach envisages the genetic manipulation of either α‐Syn or other PD‐associated genes like LRRK2, PINK1, Parkin, or DJ‐1 (Jagmag et al., 2016). However, the majority of PD cases do not have a clear genetic origin but are considered idiopathic (for a review on PD model limitations see Potashkin et al., 2010). Moreover, in most cases, the disease is modeled in young or, at best, in young adult animals. For a more precise modeling of the disease, it would be crucial to carry such studies in the context of old animals which is the natural environment for neurodegenerative diseases. Teleost fishes like Medaka and Zebrafish have also been utilized to model PD with similar approaches (Flinn et al., 2013; Matsui et al., 2009, 2013; Uemura et al., 2015), thus showing the same limitations.

The teleost *Nothobranchius furzeri* (Killifish) is the vertebrate with the shortest captive lifespan and has emerged as a valuable experimental model for aging research. This species replicates the conserved aging hallmarks of mammals and in particular shows age‐dependent gliosis (Tozzini et al., 2012), activation of immune response and spontaneous protein aggregation in neurons (Kelmer Sacramento et al., 2020), making it a particularly valuable model for brain aging.

In 2019, Matsui et al. (2019) described in a new captive strain of *N. furzeri* a spontaneous neurodegeneration in *N. furzeri* that shares some aspects of PD. In their work, they observed an age‐dependent α‐Syn accumulation associated with the formation of Synuclein aggregates and age‐dependent neuronal loss in the LC and in a diencephalic dopaminergic population (*posterior tuberculum*) that project to the subpallium and is considered a putative homolog of mammalian midbrain dopaminergic neurons (Kaslin & Panula, 2001; Rink & Wullimann, 2001).

Given the great possible significance of this report, we set to assess neurodegeneration of noradrenergic and dopaminergic neurons in other two, well‐established, wild‐type strains of *N. furzeri*.

## 2 RESULTS

In the present study, we analyzed individuals from the *N. furzeri* population MZCS‐222 that belongs to the same genetic clade of the population MZCS‐24, presenting noradrenergic and dopaminergic neurons age‐dependent reduction (Matsui et al., 2019), and the population MZM‐0410 we have used in our previous studies. This population was collected later than MZM‐0410 and is therefore likely to be more genetically heterogeneous and closer to the wild population. The median lifespan of this strain in captivity is around 6 months (Žák & Reichard, 2020).

**FIGURE 1:**
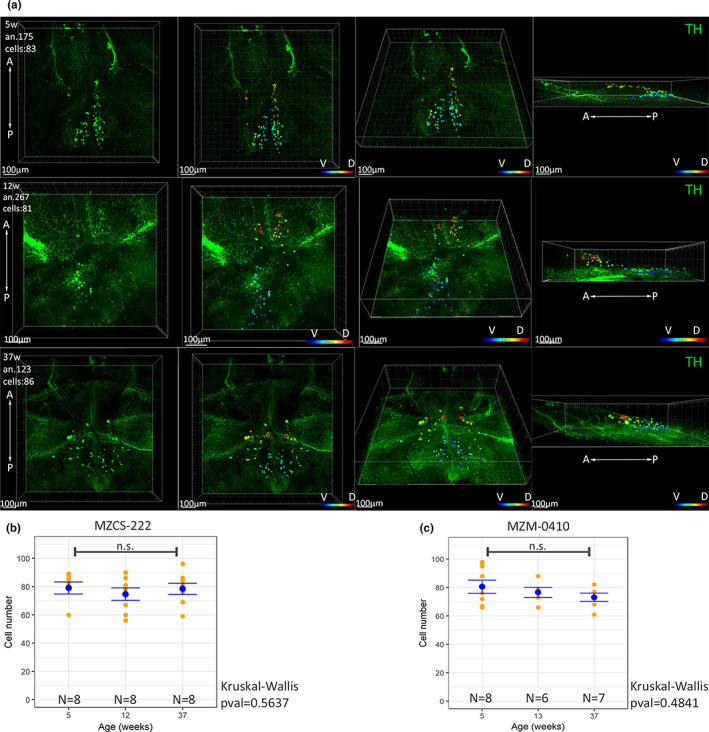
Assessment of neurodegeneration of TH+ cells in p*osterior tuberculum*. (a) Representative images of 3D reconstructions and cell counting in the *posterior tuberculum* nuclei of a young (5 weeks old), adult (12 weeks old), and old (37 weeks old) *N. furzeri*. A, anterior; D, dorsal; P, posterior; V, ventral. The color code indicates the depth of the cells in the reconstructed volume. (b) Cell counts of *posterior tuberculum* nuclei in young, adult, and old animals belonging to the strain MZCS‐222. Each yellow dot represents a single animal and the blue dot the mean of the group and the error bar the SEM. Statistically significant difference was assessed by Kruskall–Wallis test, n.s. indicates *p* > 0.05. (c) Cell counts of *posterior tuberculum* nuclei in young, adult, and old animals belonging to the strain MZM‐0410. Each yellow dot represents a single animal and the blue dot the mean of the group and the error bar the SEM. Statistically significant difference was assessed by Kruskall–Wallis test, n.s. indicates *p* > 0.05

We set to quantify the total number of TH+ cells in the *locus coeruleus* (LC) and in the *posterior tuberculum* (hypothalamus) in young (5 weeks), adult (12 weeks), and old (37 weeks) animals to assess the presence of possible neurodegeneration. The size of the brain increases during this period and the spatial distribution of cells may vary between animals of different ages (Figure 1a and Video S3). In order to visualize and count all TH+ cells in the region of interest, we clarified the brain using the Sca/eS procedure (Hama et al., 2015, 2016) and reconstructed all the different TH+ nuclei of *N. furzeri* in their entire 3D extent by scanning through the transparent brain (Figures S1 and S2).

The *N. furzeri posterior tuberculum* contains two clearly separated populations of TH+ cells (Figure 1a, Video S3), one comprised of larger and more anteriorly and dorsally located cells and one comprised of smaller and more posteriorly and ventrally located cells. These two nuclei are very similar to the nuclei 12 and 13 described in the zebrafish *posterior tuberculum* using TH immunofluorescence (Sallinen et al., 2009) and are proposed as homologous to the A‐9 A‐10 mammalian dopaminergic cluster comprising the *substantia nigra* (Kaslin & Panula, 2001; Rink & Wullimann, 2001). We counted all cells in these two populations using whole‐brain reconstruction (Figure 1a, Video S3) and could not detect a significant difference in their numbers between young, adult, and old animals in either of the two strains we analyzed (Figure 1b,c).

**FIGURE 2:**
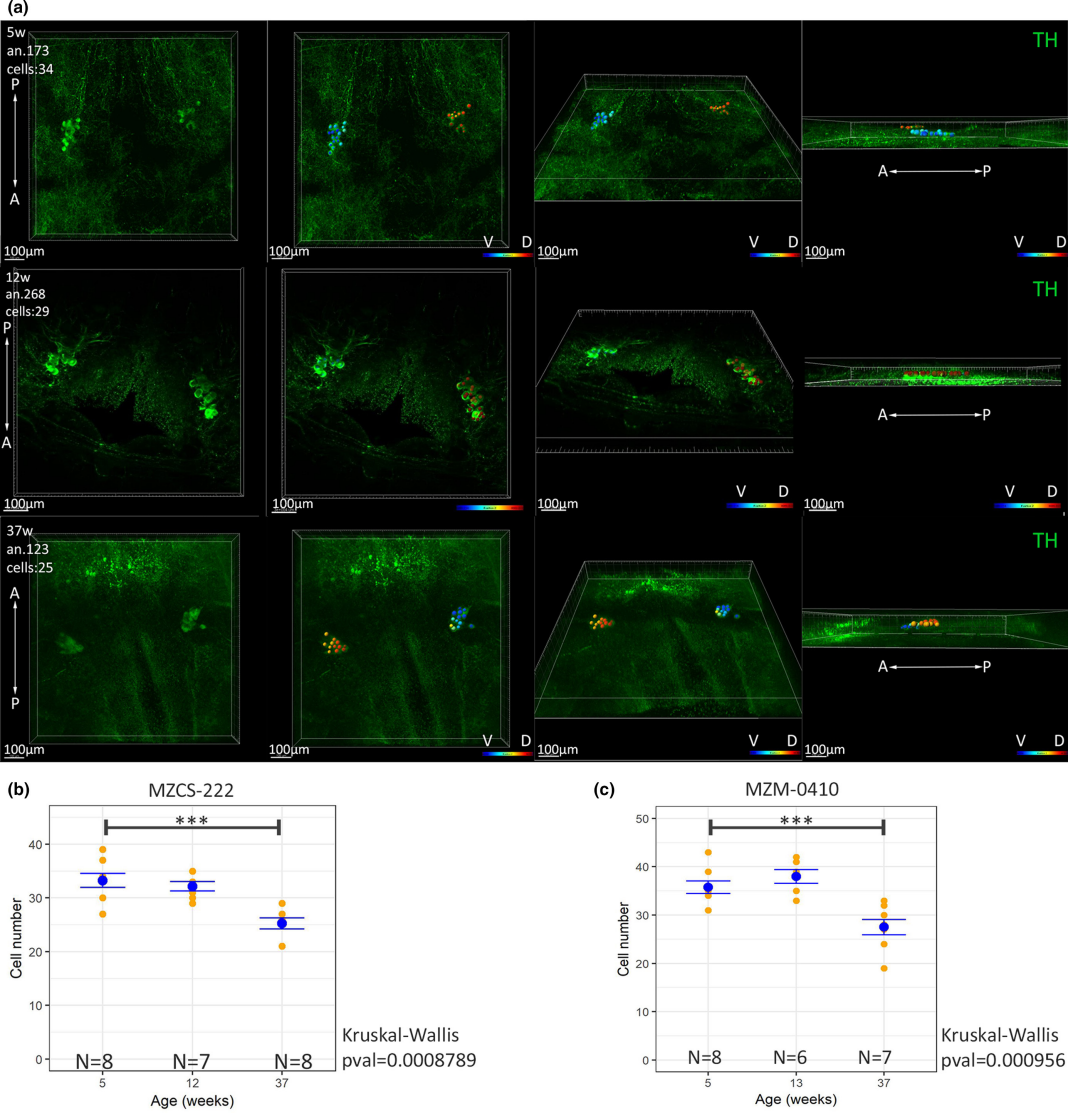
(a) Assessment of neurodegeneration pf TH+ cells of *locus coeruleus*. Representative images of a 3D reconstruction and cell counting of LC of a young (5 weeks old), adult (12 weeks old), and old (37 weeks old) *N. furzeri*. A, anterior; D, dorsal; P, posterior; V, ventral. The color code indicates the depth of the cells in the reconstructed volume. (b) Cell counts of LC nuclei in young, adult, and old animals belonging to the strain MZCS‐222. Each yellow dot represents a single animal and the blue dot the mean of the group and the error bar the SEM. Statistically significant difference was assessed by Kruskall–Wallis test, *** indicates *p* < 0.001. (c) Cell counts of LC nuclei in young, adult, and old animals belonging to the strain MZM‐0410. Each yellow dot represents a single animal and the blue dot the mean of the group and the error bar the SEM. Statistically significant difference was assessed by Kruskall–Wallis test, *** indicates *p* < 0.001

We counted also all cells present in the *locus coeruleus*, a nucleus affected during the first stages of Parkinson's disease (Figure 2a, Video S4). In this case, we could not detect a significant difference in cell numbers between 5‐ and 12‐week animals and a modest but significant reduction between 5 and 39 weeks of ~30% in both strains (Figure 2b,c). Pictures representative of the 3D reconstructions analyzed in the present study are presented in Figure S5 for hypothalamic nuclei and in Figure S6 for *locus coeruleus* cells.

**FIGURE 3:**
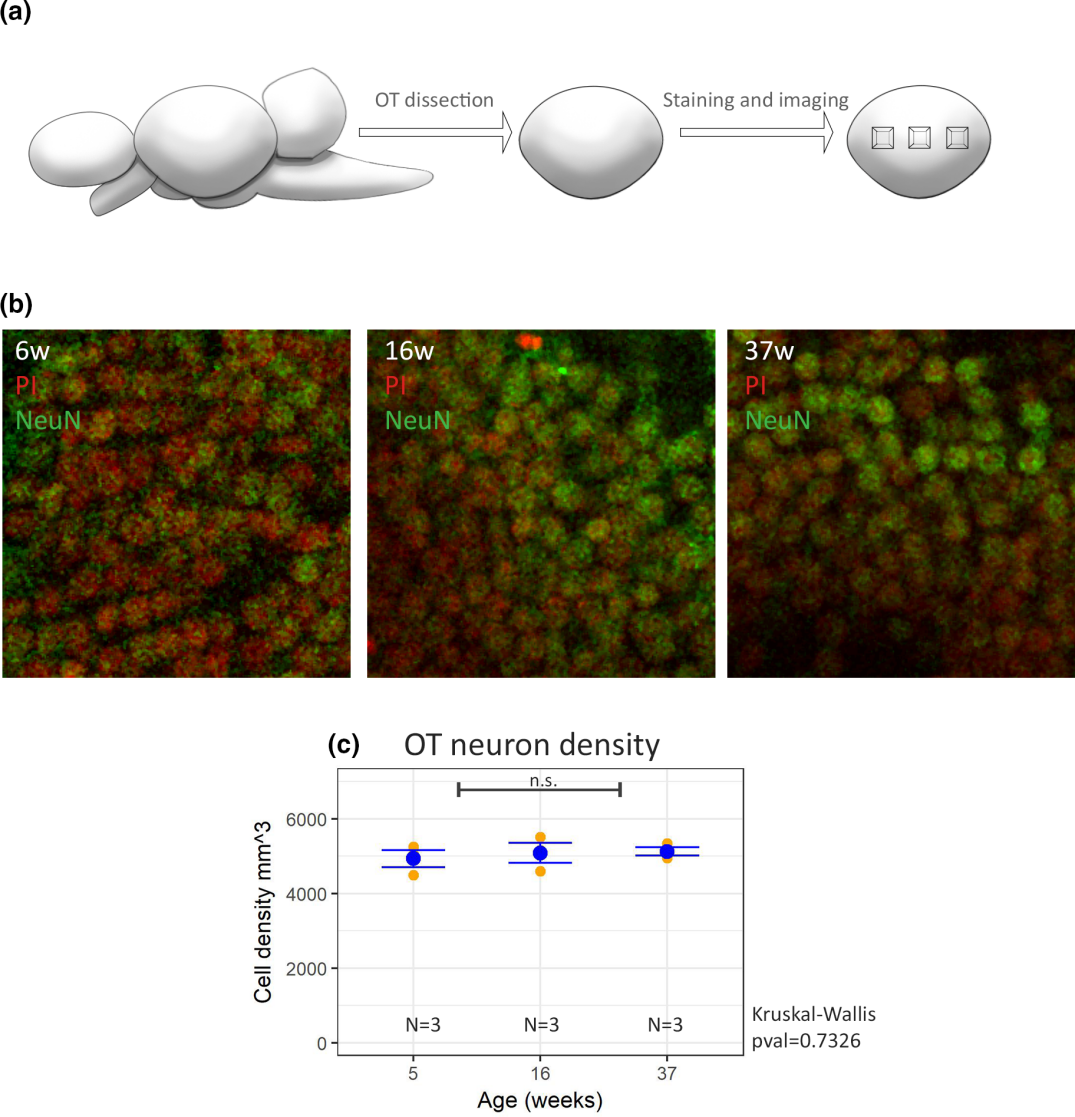
Analysis on neuronal density in the o*ptic tectum* of *N. furzeri*. a) Schematic representation of data acquisition process for *optic tecum* neuronal density. Starting from *N. furzeri* brain, we dissected the *optic tectum*, we then stained, clarified, and imaged three areas per animal. Further details regarding the procedure can be found in “Materials and Methods”. (b) Representative z‐planes from the stacks utilized to perform 3D reconstructions and counting of NeuN (green)—propidium iodide (PI, red) double positive cells in animals at 6, 16, and 37 weeks of age. (c) Plot of the estimated number of neurons per mm^3^ in the *optic tectum* at different ages. Each yellow dot represents a single animal, the blue dot represents the mean of the group and the error bar the SEM. Statistically significant difference was assessed by Kruskall–Wallis test, n.s. indicates *p* > 0.05

*N. furzeri* brain grows throughout adult life (Tozzini et al., 2012). To compare the age‐dependent dynamics of the TH+ populations with a neuronal population that is expected to increase in numerosity, we decided to analyze the optic tectum. This structure contains a well‐defined germinal layer and a continuous generation of neurons takes place during adult life that is coupled with an increase in its total area (Tozzini et al., 2012). We quantified neuronal density in the optic tectum of young, adult, and old animal stained with NeuN (a pan‐neuronal marker) and Propidium Iodide (Figure 3b), by using the same clearing, 3D reconstruction and counting method utilized for the counting of TH positive cells (Figure 3a). We found that neuronal density in a vertical column remains constant through the assessed ages (Figure 3c). So, the increase of the *optic tectum* size is not associated with thinning and must result in an increase of total neuronal numbers.

**FIGURE 4:**
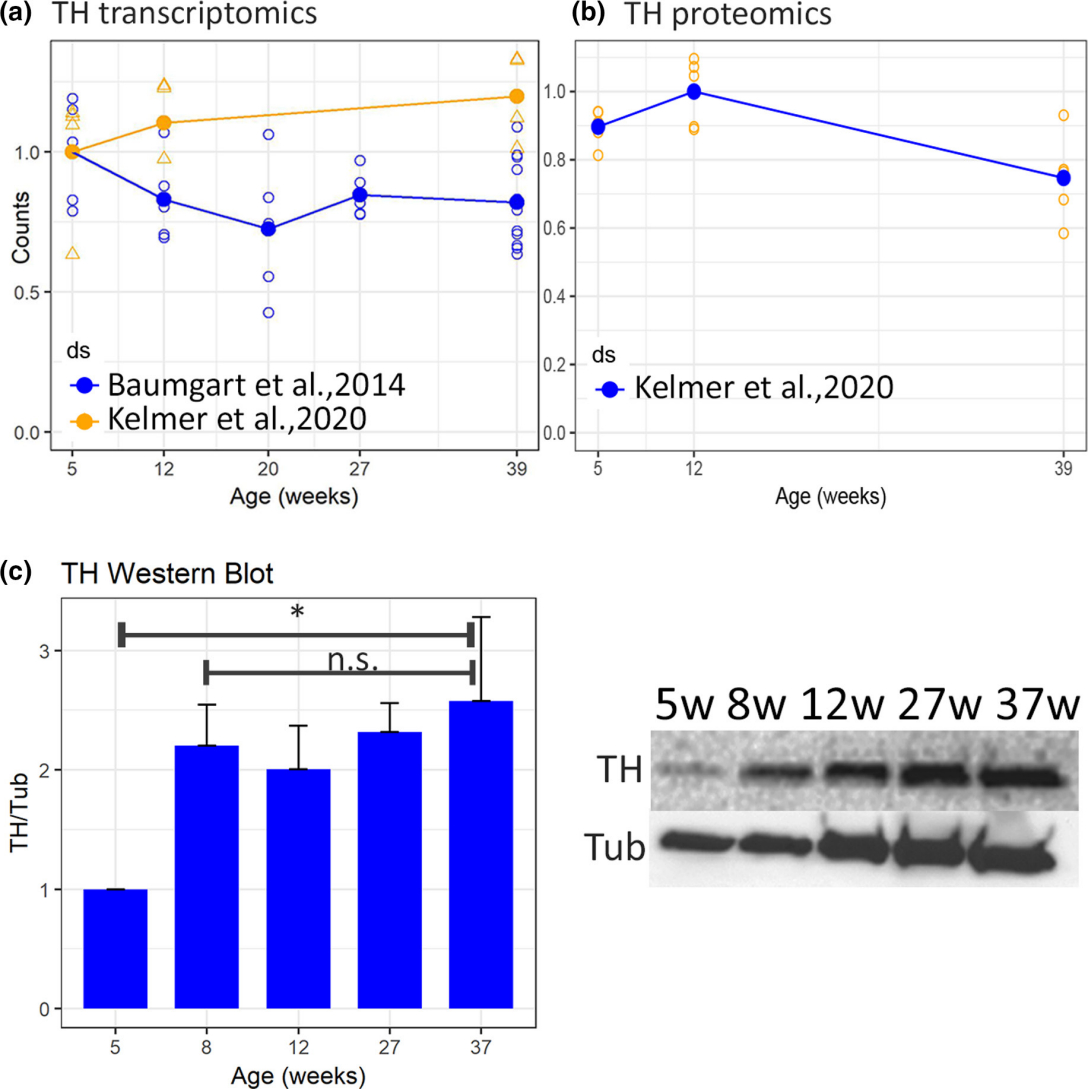
Quantification of TH expression. (a) Quantification of TH mRNA expression. Two independent RNA‐seq datasets (from Baumgart et al., 2014, and Kelmer Sacramento et al., 2020) were analyzed. (b) Analysis of TH protein expression by mass spectrometry. We assessed a proteomic dataset (from Kelmer Sacramento et al., 2020). Each open dot represents a single animal and the solid dot the mean of the group and the error bar the SEM. To assess the statistical significance of age‐dependent regulation, we calculated spearman correlation with age. In both cases *p* > 0.05. (c) Images and quantification of TH protein expression by Western blot of pooled samples. Complete blots and individual sample experiment are reported in Figure S7. A total of four samples per each age were pooled and analyzed. The intensity of TH band was normalized with respect to the tubulin band. To assess statistical significance, we calculated Pearson's correlation coefficient of the expression with age and respective *p* value, the data are expressed as mean ± SEM

To assess whether TH showed a reduction of expression at mRNA or protein level, we interrogated two independent public databases of RNA‐seq (Figure 4a) and one independent database of mass‐spectrometry based proteomics of *N. furzeri* brain aging in the MZM‐0410 strain (Figure 4b). We could not detect down‐regulation of TH neither at the transcript‐ nor at the protein level. We also performed Western blot analysis for both pooled‐ and single‐animal samples of the MZCS‐222 strain, and also in this case, we could not detect a down‐regulation of TH in the whole brain (Figure 4c, Figure S7) and rather obtained some support for an increase of TH content between 5 and 8 weeks of age.

**FIGURE 5:**
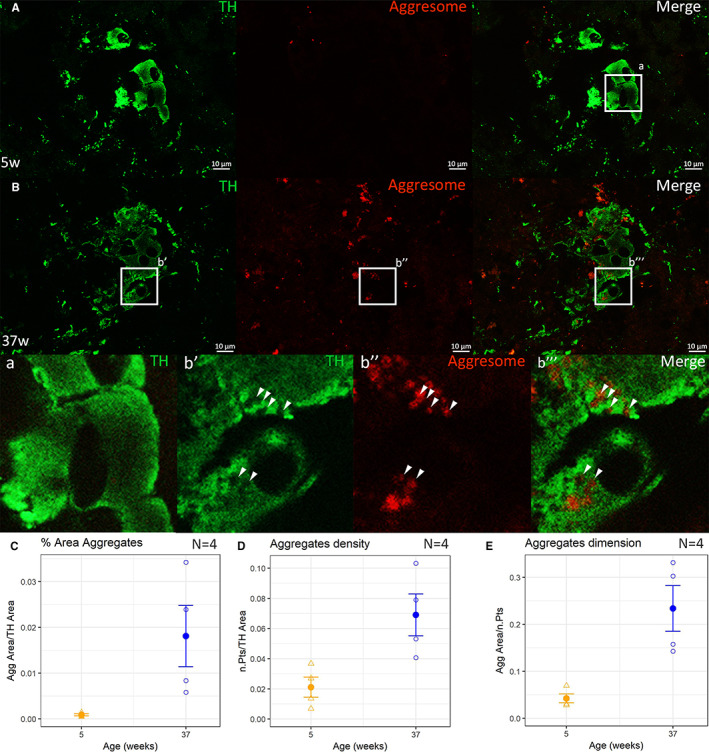
Double immunofluorescence for TH (green) and aggregates (Proteostat Aggresome dye, red) in *locus coeruleus* cells. Aggresome staining is absent in young animals (5 w, panel A) and becomes visible in old animals (37 w, panel B). In particular, aggregates are mainly located in vacuolized areas of cytoplasm devoid of TH staining (panels, b’, b”, b”’) that are not present in young animals (panel a). The area (panel C), the number (panel D), and the dimension (panel E) of the aggregates all increased with aging. To test significance two tailed t‐test was used, * indicates *p* < 0.05 and ** indicates *p* < 0.01, the data are expressed as mean ± SEM. Scale bars correspond to 10 μm.

In PD, neurodegeneration is strictly associated with the formation of pathological aggregates. To check whether this could be the case also for the neurodegeneration observed in *N. furzeri*, we investigated the presence of protein aggregates (aggresomes) in LC neurons from old (37 weeks) and young (5 weeks) fish. The Proteostat dye revealed a punctate staining in TH+ neurons from old, but not young fish (Figure 5A,B). This difference was apparent by simple visual inspection and was also confirmed by quantitative analysis (Figure 5C–E). The staining of aggresomes in LC neurons showed a peculiar localization, as protein aggregates were clearly visible in cytoplasmatic areas devoid of TH staining, indicating that they are contained within cytoplasmic vacuoles (Figure 5B b’–b”’).

## 3 DISCUSSION

In our paper, we report an age‐dependent degeneration of noradrenergic neurons in the LC, but not of dopaminergic neurons in the *posterior tuberculum*, a brain region supposed to be the homolog of mammalian A8 and A9 dopaminergic populations. To precisely assess neurodegeneration, we decided to implement a whole‐brain clarification technique to count all cells in the nuclei of interest.

Our results indicate an absence of neuronal loss in the *posterior tuberculum* nuclei between young and old animals of the strains MZCS‐222 and MZM‐0410 as opposed to the ~42% reduction observed in the MZCS‐24 strain (Matsui et al., 2019).

However, fish brains grow throughout life due to the presence of continuous neurogenesis (Kaslin et al., 2008). To what extent adult neurogenesis increases cell number or rather represents a turnover process is not known in detail, as total neuronal numbers were not quantified in fish of different ages. Brain growth may indeed be the result of increased cell size and/or neuropil rather than addition of new neurons. We specifically quantified neuronal density in the optic tectum, an area with a defined germinal layer (Tozzini et al., 2012) and whose flat structure and concentration of neurons in the subventricular gray zone facilitates neuronal counting. The stability of neuronal density despite an increase in surface clearly indicates that adult neurogenesis results in an increase in total neurons number. TH‐positive cells of the *posterior tuberculum* instead do not show an increase of cell number postnatally and remain constant after the animal reaches maturity (i.e., 5 weeks of age) indicating absence of adult neurogenesis for this population. This result is consistent with the observation that in zebrafish the optic tectum can regenerate new neurons after lesion (Lindsey et al., 2019) while neurons of the posterior tuberculum do not show constitute neurogenesis and are not replaced after lesion (Caldwell et al., 2019).

In the *locus coeruleus*, we could not detect a significant reduction between young and adults (5 vs 12 weeks of age) in either of the two strains we analyzed, but a significant reduction between 5 and 39 weeks. The amplitude of the reduction is, however, of ~30% in both our strains, while previous studies (Matsui et al., 2019) reported in the strain MZCS‐24 a reduction present already between 5 weeks and 12 weeks of age, and progressing at later stages, amounting to a total of ~80%.

The results of our analysis seem in contrast with the observations reported by Matsui et al. (2019) who reported loss of noradrenergic and dopaminergic neurons in *N. furzeri* and a down‐regulation of TH expression in homogenates from the whole brain in the strain MZCS‐24, in their paper assessing the validity of *N. furzeri* as a model of synuclein‐dependent neurodegeneration.

Two main factors may account for the discrepancies between our observations and previous reports conducted in the strain MZCS‐24. A first intriguing possibility is the existence of genetic differences between the strains, even if the population MZCS‐222 and MZM‐0410 we analyzed here and the population MZCS‐24 analyzed previously (Matsui et al., 2019) are part of the same genetic clade. The distance between the collection points is around 50 km but *N. furzeri* is characterized by an extreme genetic population structuring and genetic differences are detectable even between populations within that order of geographic separation (Bartáková et al., 2013). It is therefore possible that the strain MZCS‐24 harbors specific variants that increase the susceptibility of dopaminergic neurons. The second possible explanation for this discrepancy is of methodological nature. We cleared the entire brain and reconstructed the nuclei of interest in their entire 3D extent instead of counting cells in thick sections. *N. furzeri* brain grows considerably during adult life. It is possible that counting from thick sections may target the entire TH+ population in young animals, but may undersample the same cells in older, larger animals. Indeed, our 3D reconstructions show that dopaminergic neurons of the *posterior tuberculum* are more widespread in brains from old fish.

To assess down‐regulation of TH expression, we used Western blot in the strain MZCS‐222 and non‐targeted approaches such as RNA‐seq and mass‐spectrometry based proteomics in the strain MZM‐0410. We could not find any evidence for a down‐regulation of TH expression. This result is concordant with the small amplitude of neurodegeneration observed in our strains: The presence of a large number of TH+ cells in brain regions other than the *locus coeruleus* (e.g., olfactory bulb and vagal nuclei) suggests that a specific loss of a few cells with widespread projections cannot impact on the total abundance of TH in the brain.

Neurodegeneration is associated with accumulation of the pathogenic post‐translational modification pS129 forming pathological aggregates in the form of Lewy bodies and Lewy neurites.

It should be noted that we do not have evidence for formation of Lewy bodies or large aggregates.

In summary, our results confirm age‐dependent neurodegeneration of LC neurons, a condition reminiscent of the presymptomatic stage of PD indicating that *N. furzeri* could be used in the future to identify modifying factors for age‐dependent neurodegeneration, and open the intriguing possibility that natural genetic variation may influence the susceptibility of dopaminergic neurons.

## 4 MATERIAL AND METHODS

### 4.1 Fish maintenance and sampling

MZM‐222 were hatched and housed locally in a Tecniplast Zebtech system with automatized water flow, pH, and salinity control. All the animals were hatched, fed, and maintained as described in detail in (Terzibasi et al., 2008).

The protocols of fish maintenance were carried out in accordance with all animal use practices approved by the Italian Ministry of Health (Number 96/2003a) and the local animal welfare committee of the University of Pisa.

Fish of the MZM0410 line were bred at the Leibniz Institute on Aging, Fritz Lipmann Institute, Jena. Procedures for fish breeding, husbandry and euthanasia were performed in accordance with the rules of the German Animal Welfare Law and approved by the Landesamt für Verbraucherschutz Thüringen, Germany.

Eggs were maintained on wet peat moss at room temperature in sealed Petri dishes. When embryos had developed, eggs were hatched by flushing the peat with tap water at 16–18°C. Embryos were scooped with a cut plastic pipette and transferred to system tank. Fry were fed with newly hatched *Artemia nauplii* for the first 2 weeks and then weaned with finely chopped *Chironomus* larvae. The system water temperature was set at a constant 27°C.

At the desired age, fish were sacrificed via anesthetic overdose (Tricaine, MS‐222), in accordance with the prescription of the European (Directive 2010/63/UE) and Italian law (DL 26/04‐03‐2014), and the brain was immediately extracted under a stereomicroscope and fixed overnight in a solution of PFA 4% in PBS.

Samples used for Sca/eS procedure were then gradually dehydrated by sequential steps of incubation in solutions with growing EtOH concentration (25%, 50%, 75%) and finally stored at −20° in EtOH 90% until use.

Samples used for immunofluorescence were instead incubated overnight in sucrose 30% at 4°C and the day after were included in cryo‐embedding medium (Tissue‐Tek® O.C.T., Sakura Finetek). Serial slices of 25 μm of thickness were then cut using a Leica cryostat and collected on Superfrost plus slides® (Thermo scientific).

### 4.2 Sca/eS procedure

Sca/eS technique is a clarification technique described in (Hama et al., 2015, 2016).

We followed the principal steps of the AbSca/e technique, which is a subtype of Sca/eS thought especially to combine immunofluorescence and tissue clarification, adapting some of the steps to the considerably smaller size of *Nothobranchius furzeri* brains. All the steps and the solutions used for this procedure are summarized in Tables 1 and 2.

**TABLE 1:** Composition of the solution used for the Sca/eS protocol

Ingredients	Sca/eA2	Sca/e B4(0)	Sca/eS0	Sca/eS4	AbSca/e	AbRinse
D‐(−)‐sorbitol (w/v%)	–	–	20	40	–	–
Glycerol (w/v)%	10	–	5	10	–	–
Urea (M)	4	8	–	4	0.33	–
Triton‐X‐100 (w/v)%	0.1	–	–	0.2	0.5	0.05
Methyl‐β‐cyclodextrin (mM)	–	–	1	–	–	–
γ‐Cyclodextrin (mM)	–	–	1	–	–	–
N‐acetyl‐L‐hydroxyproline (w/v)%	–	–	1	–	–	–
DMSO (w/v)%	–	–	3	25	–	–
PBS	–	–	1x	–	–	0.1x
BSA (w/v)%	–	–	–	–	–	2.5

*Note*: The composition is taken as indicated in Hama et al., 2015.

**TABLE 2:** Sca/eS procedure

Step	Solution	Timing (approx.)	Temp.
Fixation	4% PFA	ON	4°C
Adaptation	Sca/e S0	18 h	37°
Permeabilization	Sca/e A2	36 h	37°
	Sca/e B4(0)	24 h	37°
	Sca/e A2	12 h	37°
Descaling	PBS	6 h	RT
Immunostaining	AbSca/e + primary antibody	3 days	4°
	AbSca/e	2 h (2x)	RT
	AbSca/e + secondary antibody	18 h	4°
Wash	AbSca/e	6 h	RT
Rinse	AbRinse	2 h (2x)	RT
Refixation	4% PFA	1 h	RT
Wash	PBS	1–2 h	RT
Clearing	Sca/e S4	18 h	37°
Mounting	Sca/e S4		4°

*Note*: This is the procedure we adapted from Hama et al., 2015 for clearing and staining of *Nothobranchius furzeri* brains. In the table are reported the solution required for each step with the time and temperature of incubation.

Abbreviations: ON, overnight; RT, room temperature.

Briefly, the samples were re‐hydrated via incubation in solutions containing decreasing concentrations of ethanol (90% → 75% → 50% → 25% → PBS) and adapted with the S0 solution for 18 h at 37°C. Samples were then permeabilized with sequential incubations in A2‐B4(0)‐A2 solutions at 37°C. After permeabilization, the samples were de‐Sca/ed through incubation in PBS for 6 h at RT followed by incubation with primary antibody in AbSca/e solution for 3 days at 4°C (TH, clone EP1533Y, ab75875, Abcam or NeuN, ab177487, Abcam, both used at a concentration of 1:500), rinsed twice in AbSca/e for 2 h each and incubation with secondary antibody (Alexa Fluor 488 goat anti‐rabbit; Invitrogen ab150077, 1:500) for 18 h at 4°C. The samples were then rinsed for 6 h in AbSca/e and subsequently in AbRinse solution twice for 2 h. For samples stained with NeuN, we proceeded also to stain nuclei with a solution of 1:500 Propidium Iodide (P4864, Sigma) in PBS for 10 min and performed an ulterior wash in AbRinse for 2 h. After a re‐fixation step in PFA4% for 1 h and a rinse in PBS for another hour, the samples were finally clarified in Sca/e S4 for 18 h at 37° and maintained in Sca/e S4 at 4° until imaging. The Sca/eS4 solution was also used as an embedding medium during imaging.

### 4.3 Immunofluorescence and Proteostat™ Aggresome staining

We performed immunofluorescence experiments on horizontal cryo‐sections of 25 μm thickness and proceeded as previously described (Tozzini et al., 2012). Briefly, we washed the sections in PBS to remove the cryo‐embedding medium; then, we performed an acid antigen retrieval step (Tri‐sodium citrate dehydrate 10 mM, tween 0,05%, pH 6). Afterward, we stained the sections with Aggresome (ProteoStat™ Aggresome Detection Kit, Enzo Life Sciences Inc.; for more details see Shen et al., 2011) as follows: We applied a solution 1:2000 of Aggresome dye in PBS for 3 min, rinsed in PBS and left the sections immersed in 1% acetic acid 40 min for de‐staining. We applied blocking solution (5%BSA, 0,3% Triton‐X in PBS) for 2 h, then the primary antibody at proper dilution in a solution of 1% BSA, 0,1% triton in PBS, and incubated the samples overnight at 4°. After rinsing in PBS, the following day secondary antibody was applied at a 1:400 dilution in the same solution used for the primary antibody. After 2 h, slides were rinsed 3 times with PBS and mounted with a specific mounting medium added with nuclear staining (Fluoroshield DAPI mounting, Sigma‐Aldrich). The antibodies utilized were thyroxine hydroxylase (Rabbit Monoclonal, ab75875, Abcam), Alexa Fluor 568 Goat anti‐Mouse (A11004), and Alexa Fluor 488 Goat anti Rabbit (A11008) (Invitrogen).

### 4.4 AbSca/e samples images acquisition and processing

To analyze the AbSca/e treated samples, we acquired images on a Leica Ire2 or a Zeiss LSM900 Airyscan confocal microscope, using a 20x objective.

We acquired 1024 × 1024 pixel sequential focal planes along the z‐axis, at a distance of 1.5 μm each, of the regions of interest and then realized and processed the 3D reconstruction utilizing the Bitplane Imaris™ software. First, we applied median filtering to reduce background and increase the quality of the stack; then, we adjusted the tone curve accordingly to the characteristics of the imaged sample.

To count the cells, we utilized the Imaris™ “Ortho Slicer” function to optically isolate portions of the z‐stack and the function “Spots” to manually count of every TH‐ or NeuN‐positive cell.

### 4.5 Optic tectum cell density analysis

To obtain information regarding the density of neurons in the *Optic tectum* of young (5 weeks), adult (16 weeks), and old (37 weeks) *N. furzeri* samples, we acquired confocal images of three areas per individual with a 20x objective (as described in the section *AbSca/e samples images acquisition and processing*, and shown in Figure 1a). We then proceeded by reconstructing the entire 3D extent of the cellular layer of the *Optic tectum* and counting all the positive NeuN nuclei in the cellular layer in a 3D region of interest of 513 × 513 px (corresponding to a scaled area of 160,056 μm^2^, 25% of the total picture area) and spanning the entire subventricular gray zone. We then averaged the cell counted in the three different areas, one central and two more lateral to obtain the mean density per animal in 160,056 μm^2^. From that value, we calculated the density of cells in a mm^3^ of *Optic tectum*. The average number of cells counted per fish was 2424.

### 4.6 Aggresome analysis into the locus coeruleus

We analyzed images of *locus coeruleus* acquired at a Zeiss AxioScan microscope equipped with Apotome slide on a 40x magnification, all with the same exposure time and to which we applied the “Best Fit” option in ZenBlue. We analyzed four animals per age. We acquired a z‐stack of the area and then analyzed the single focal plane presenting the majority of TH staining. We used the open license software Icy (http://icy.bioimageanalysis.org/) for spot analysis. To the selected z‐plane, we applied the algorithm “Best Thresholder” (method “Otsu”) on the TH channel to isolate the *locus coeruleus* cells. Since TH is not distributed homogeneously into the cytoplasm but presents vacuolization containing aggregates, we adjusted manually the selected ROI to include areas containing aggregates. We then used the algorithm “Spot Detector” imposing to perform the analysis only in the selected ROI with the following parameters:

Preprocessing: red channel.

Detector: Detect bright spots over dark BG, or scale 2.

Region of interest: ROIFixedFromSequence.

Output: export to ROI.

The ROIs created (containing the data of both the area of TH and each single spot detected) were exported in an excel file, and the following parameters were calculated:

- Percentage of stained area: spots area/TH area.
- Mean spots abundance: number of spots/TH area.
- Mean spot dimension: spots area/number of spots.

### 4.7 Western blot

We performed Western blot experiments to quantify the expression of thyroxine hydroxylase in the brains of MZ‐222 *Nothobranchius furzeri* at various ages. The brains were extracted, immediately stored at −80°, and then homogenized for 30 seconds using GelD2 buffer with addition of protease and phosphatases inhibitors. After centrifugation for 10 min at 16,000 rpm, supernatant was taken, quantified using the BCA kit (Thermofisher) and stored at −80° for further use.

To perform the Western blot, we run 20 μg of protein extract derived from four samples per age (pooled or independently) and run them in precast gels (AnyKD Mini‐Protean TGX Gels, Biorad) for 35 min at 100 V. Afterward, the samples were blotted on a nitrocellulose membrane for 35 min at 150 V. The membranes were then imaged with a Chemidoc XRS scanner using the Quantity one Biorad software, and band intensity quantification was performed using the opensource ImageJ software. The two membranes of single animal samples were processed in parallel throughout the experiment and the acquisition phase. We analyzed four animals for each age (5, 8, 12, 27, 37 weeks).

### 4.8 RNA‐seq and proteomics data analysis

To assess TH expression, we realized graphics of RNA expression starting from RNA‐seq data derived from public cross‐sectional experiments (Baumgart et al., 2014; Kelmer Sacramento et al., 2020). We combined two RNA‐seq datasets of brain aging in *N. furzeri*: a dataset covering five time points (5, 12, 20, 27, and 39 weeks) and 5 biological replicates for each age and the second containing 4 replicates per ages: 5, 12, and 39 weeks. These ages correspond to sexual maturity, young adult, adult (as defined by a decrease in growth rate), median lifespan and old (~30% survivorship) (Baumgart et al., 2014). In total, these represent 37 different samples spanning five different ages. We first normalized the samples using the Deseq2 package in the suite R and then divided the counts of the two datasets for the average expression of their respective 5 w samples to be able to combine the datasets.

We also analyzed publicly available proteomic data (Kelmer Sacramento et al., 2020). The dataset contains five replicates for age (5, 12, 39 weeks) and is a combination of two separate experiments performed utilizing tandem mass tag and analyzing the same 12 w animals twice in two different contrasts: 39 w vs 12 w and 5 w vs 12 w., Therefore, we combined the data normalizing the protein expression dividing them for the mean value of the 12 w animals.

For both transcriptomics and proteomics data we performed statistical analysis calculating the Spearman correlation value, the p value adjusted and the FDR to assess the statistical significance of the observed expression variability.

All the analysis were performed utilizing the suite R.

## AUTHOR CONTRIBUTIONS

Sara Bagnoli and Alessandro Cellerino designed the experiments. Sara Bagnoli performed the experiments with help from Eva Terzibasi Tozzini, Baldassare Fronte, and Carlo Bibbiani. Alessandro Cellerino and Eva Terzibasi Tozzini supervised the work. Sara Bagnoli and Alessandro Cellerino wrote the paper with help from all the authors.

## FUNDING INFORMATION

The work was partially supported by the grant SNS2019 to AC and the Project “Etherna” from Fondazione Pisa.

## CONFLICT OF INTEREST

The authors declare no conflict of interest.

## Supporting information


Figure S1

Figure S2

Video S3

Video S4

Figure S5

Figure S6

Figure S7

Appendix S8

## Data Availability

Images (.tiff), IMARIS 3D reconstructions, R scripts, and data utilized for the analysis are deposited and available at the following link: https://data.mendeley.com/datasets/4njyjn4s86/draft?a=9bb9bc24‐ae3a‐420b‐ab68‐8edd7facb8a0. All data analyzed in this study are taken from the published articles Baumgart et al. (2014) and Kelmer Sacramento et al. (2020) (and in their additional files).
