# Supplementary figures and images for: Quantification of noradrenergic‐, dopaminergic‐, and tectal‐neurons during aging in the short‐lived killifish Nothobranchius furzeri
Source: Aging Cell. 2022 Aug 19;21(9):e13689. doi: 10.1111/acel.13689 (PMC9470901; doi:10.1111/acel.13689)

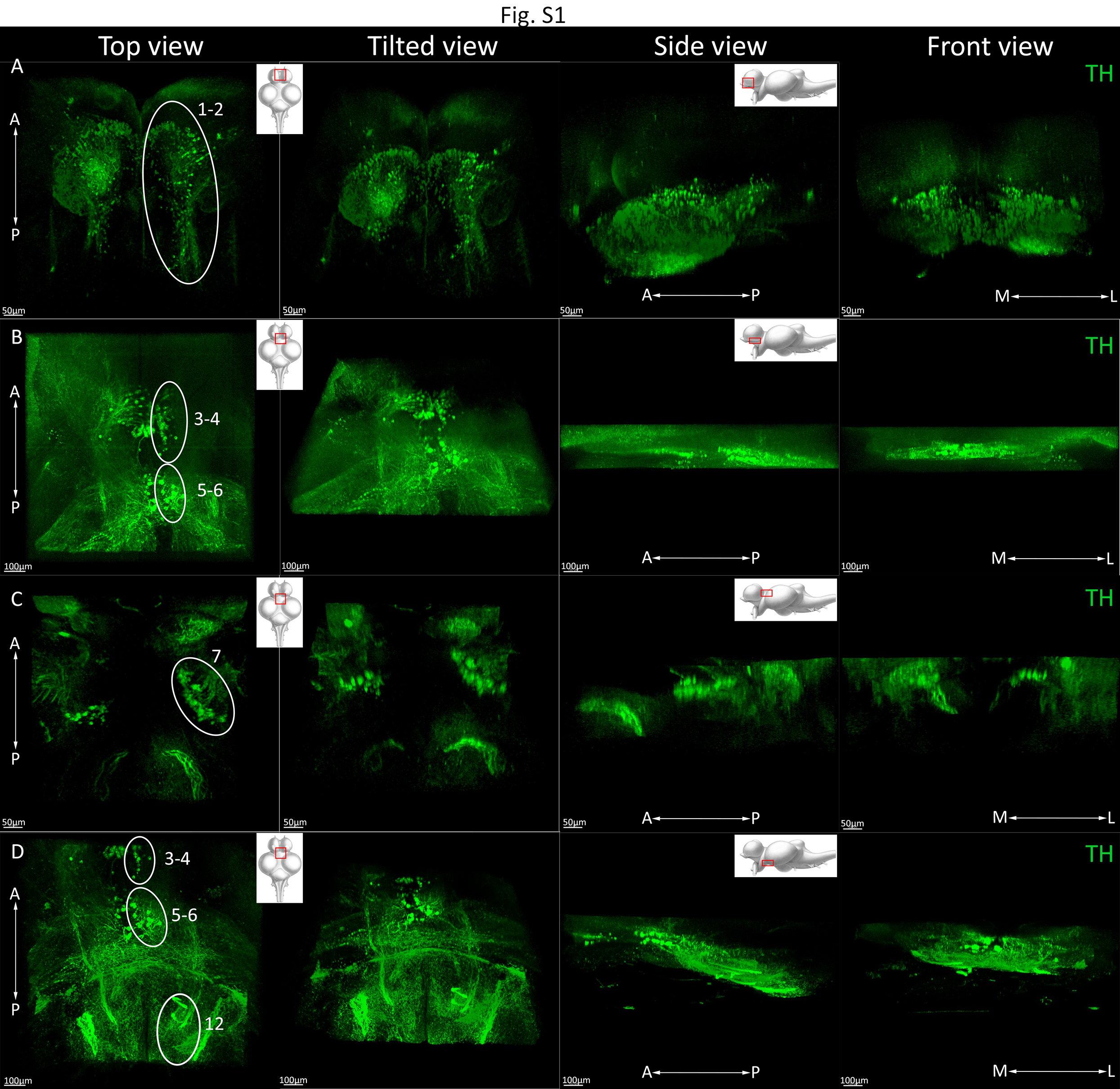

Supplement: Supplementary file 1 — Figure S1 [file ACEL-21-e13689-s007.tif]

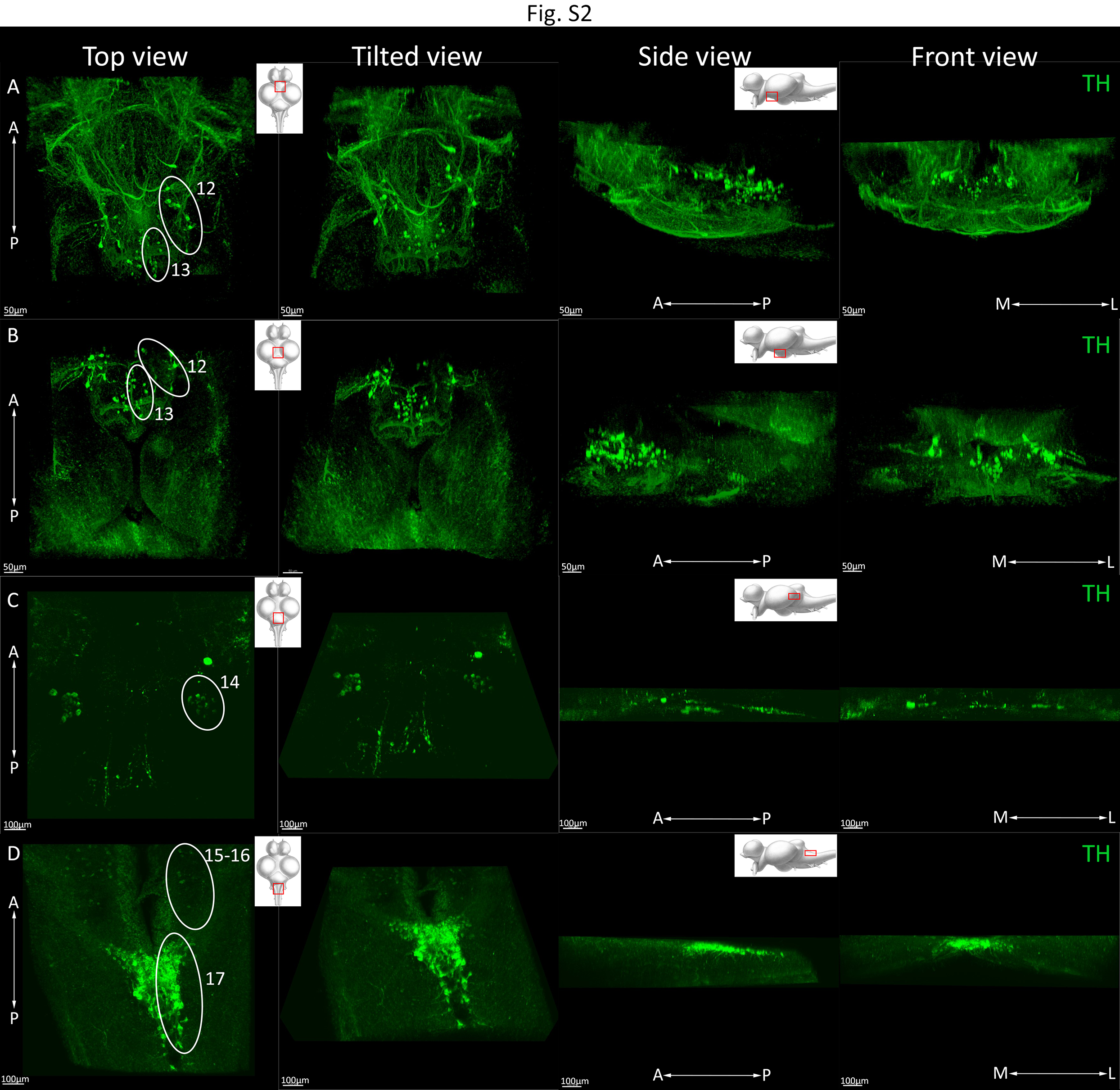

Supplement: Supplementary file 2 — Figure S2 [file ACEL-21-e13689-s006.tif]

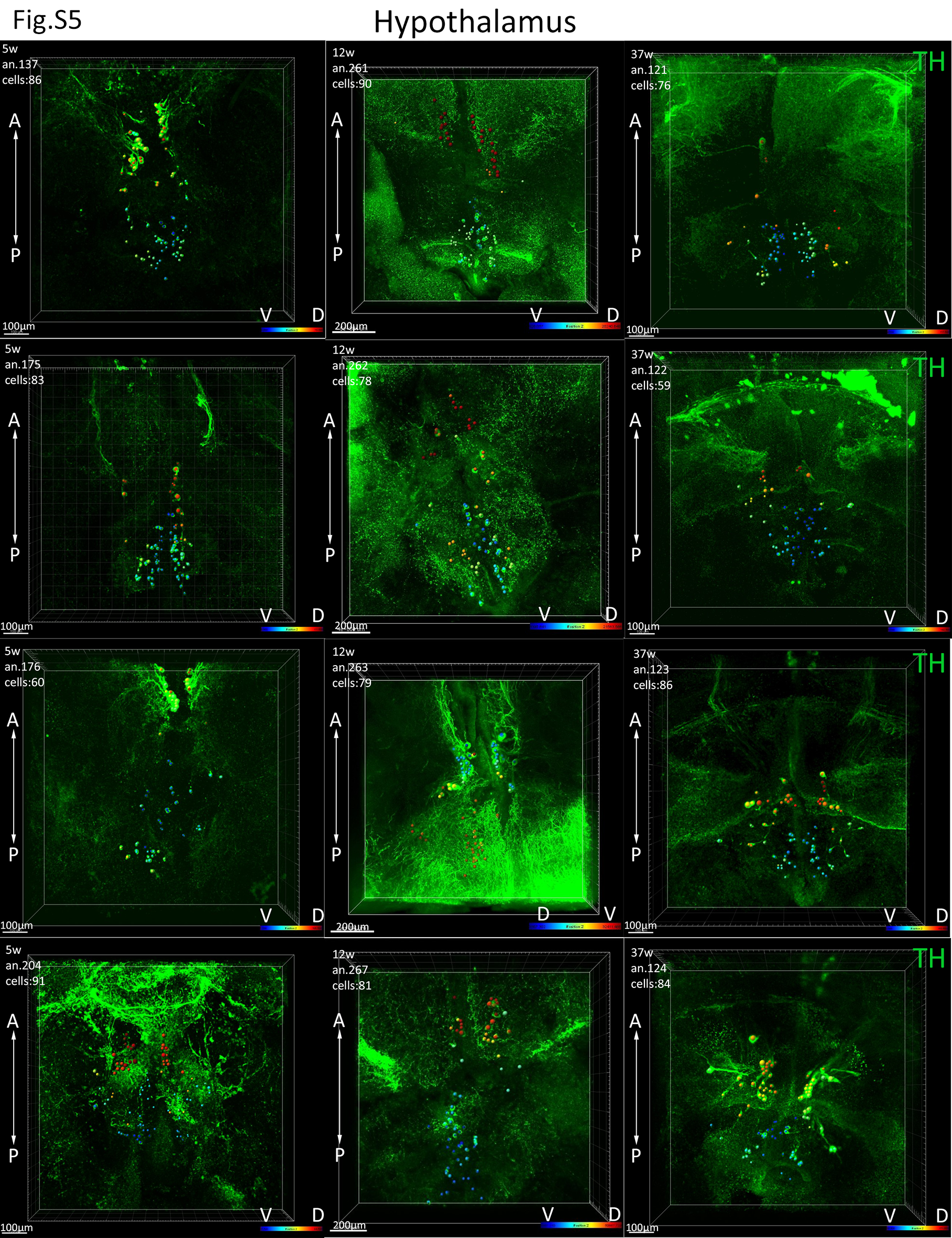

Supplement: Supplementary file 5 — Figure S5 [file ACEL-21-e13689-s003.tif]

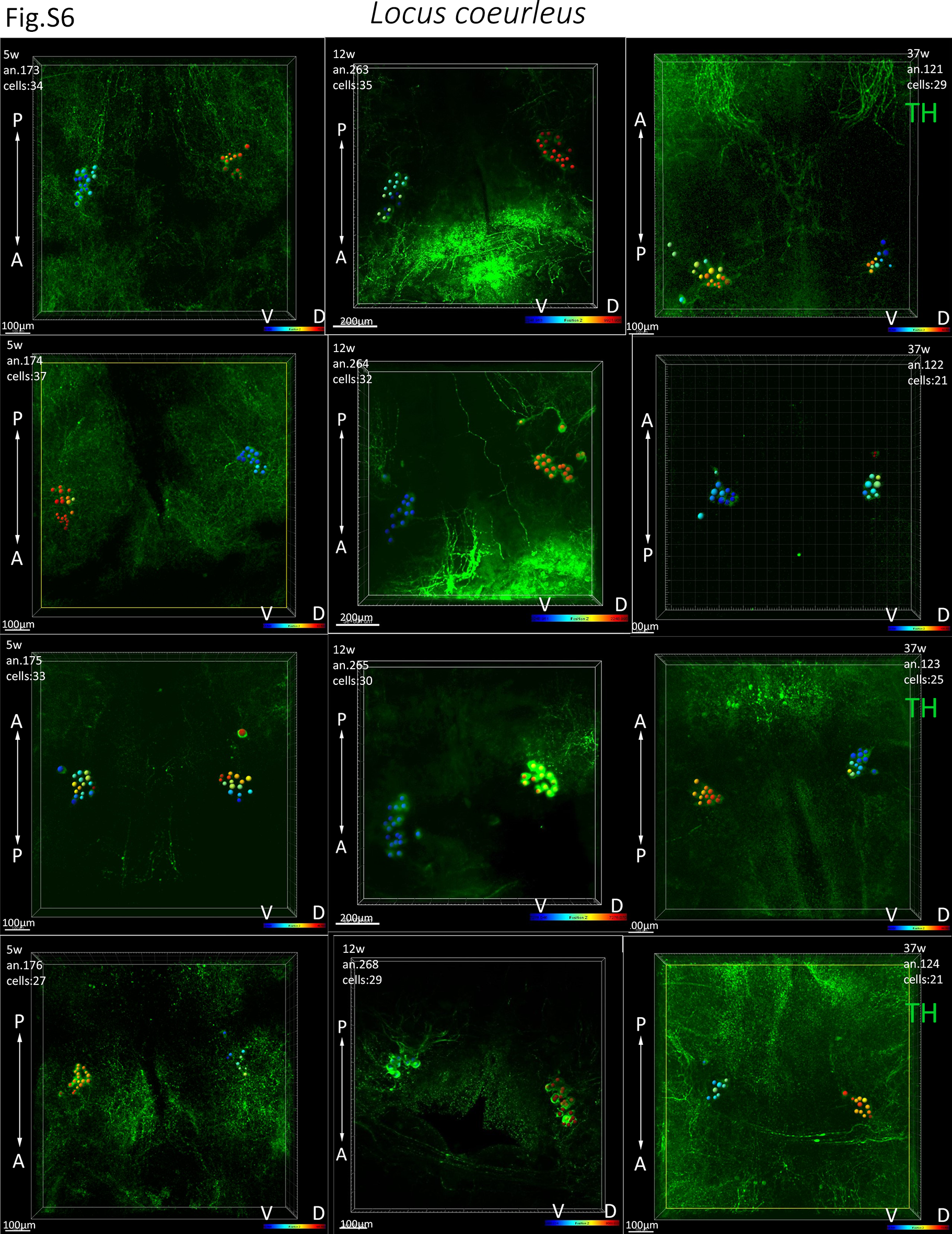

Supplement: Supplementary file 6 — Figure S6 [file ACEL-21-e13689-s002.tif]

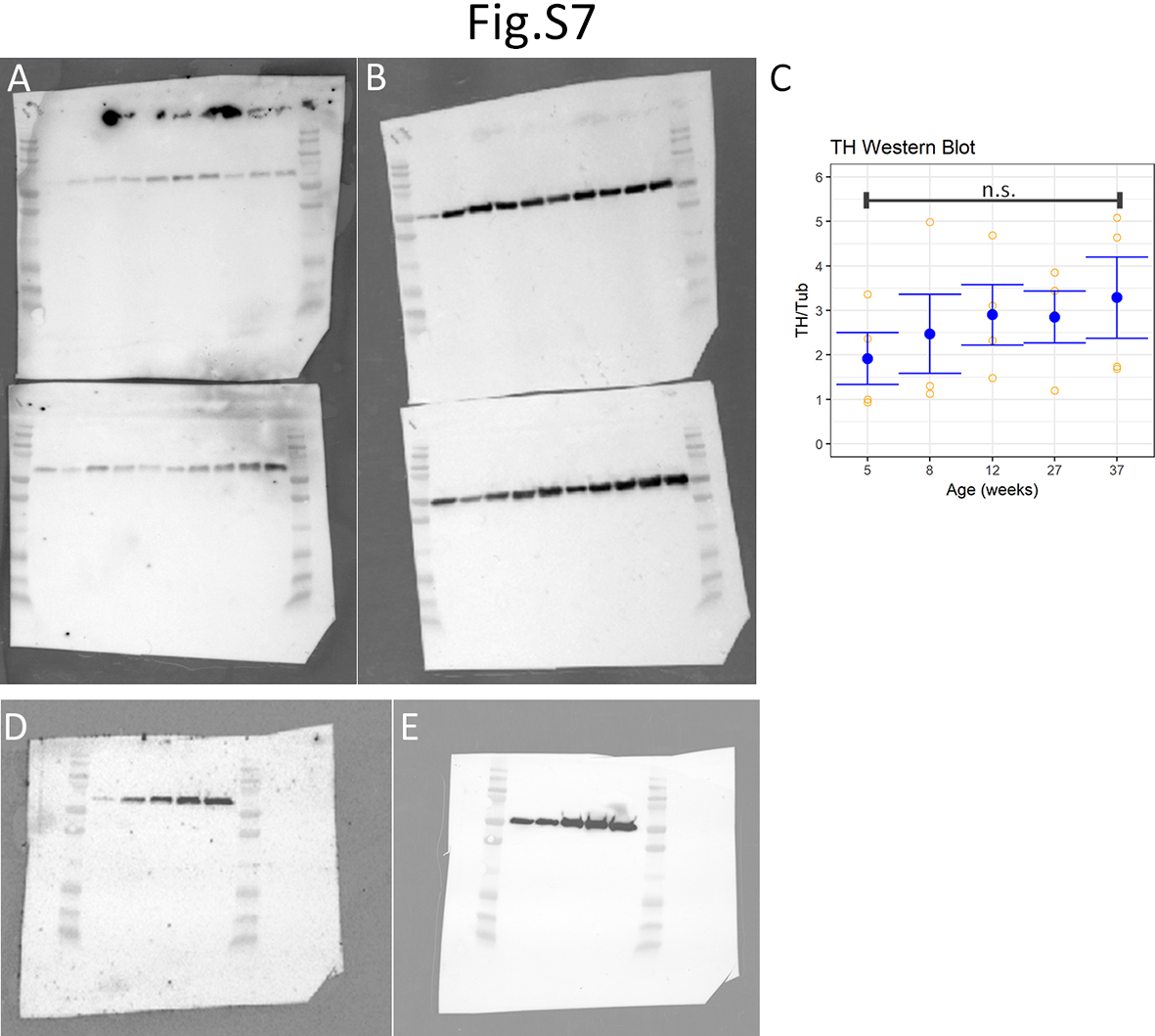

Supplement: Supplementary file 7 — Figure S7 [file ACEL-21-e13689-s008.tif]
