# Supplementary material for: Quantification of noradrenergic‐, dopaminergic‐, and tectal‐neurons during aging in the short‐lived killifish Nothobranchius furzeri
Source: Aging Cell. 2022 Aug 19;21(9):e13689. doi: 10.1111/acel.13689 (PMC9470901; doi:10.1111/acel.13689)
Supplement: Supplementary file 8 — Appendix S8 [file ACEL-21-e13689-s001.docx]

**Supplemetary Information**

Fig. S1 Localization of the TH+ nuclei in the brain of *Nothobranchius furzeri*, anterior. As the main reference for nuclei identification a Zebrafish map from Sallinen et al. was used. A) main nuclei of the olfactory bulbs and ventral telencephalon (homolog to nuclei 1-2 from Sallinen et al., 2009). B) caudal telencephalic nuclei and rostral diencephalic nuclei (homolog to nuclei 3-4 and 5-6 from Sallinen et al., 2009). C) periventricular pretectal nuclei (homolog to nuclei 7 from Sallinen et al., 2009). D) caudal telencephalic nuclei, rostral diencephalic nuclei and beginning of periventricular organ, *Posterior tuberculum* (homolog to nuclei 3-4, 5-6 and 12 from Sallinen et al., 2009). A=anterior, P=posterior, M=medial, L=lateral.

Fig. S2 Localization of the TH+ nuclei in the brain of *Nothobranchius furzeri*, posterior. As the main reference for nuclei identification a Zebrafish map from Sallinen et al. was used. A) Main diencephalic nuclei of the *Posterior tuberculum*: periventricular organ and periventricular hypothalamus (homolog to nuclei 12-13 from Sallinen et al., 2009). B) more caudal view of main diencephalic nuclei of the *Posterior tuberculum*: periventricular organ and periventricular hypothalamus (homolog to nuclei 12-13 from Sallinen et al., 2009). C) *Locus coeruleus* nuclei (homolog to nuclei 14 from Sallinen et al., 2009). D) Vagal nuclei (homolog to nuclei 15-17 from Sallinen et al., 2009).

Video S3. Comparison of 3D reconstruction of *posterior tuberculum* (hypothalamus) of young (5w) and old (37w) *N. furzeri* with cell count.

Video S4. Comparison of 3D reconstruction of *locus coeruleus* of young (5w) and old (37w) *N. furzeri* with cell count.

Fig. S5 Comparison of representative 3D reconstructions with cell count of TH+ cells in young (5w), adult (12w) and old (37w) *N. furzeri posterior tuberculum* (hypothalamus). A= anterior, P=posterior, V=ventral, D=dorsal.

Fig. S6 Comparison of representative 3D reconstructions with cell count of TH+ cells in young (5w), adult (12w) and old (37w) *N. furzeri* *locus coeruleus*. A= anterior, P=posterior, V=ventral, D=dorsal.

Fig. S7 Integral images for TH western blot. A) Integral original image for single animals Western blot of TH. B) Integral original image of Tubulin for single animals Western blot of TH. C) Quantification of the single animal Western blot. D) Integral image for TH of the pooled animal Western Blot E) Integral image of Tubulin of the pooled animal Western Blot.
